# Role of Tunneling Nanotubes in the Nervous System

**DOI:** 10.3390/ijms232012545

**Published:** 2022-10-19

**Authors:** Olga Tarasiuk, Arianna Scuteri

**Affiliations:** Experimental Neurology Unit and Milan Center for Neuroscience, School of Medicine and Surgery, University of Milano-Bicocca, 20900 Monza, Italy

**Keywords:** TNTs, neurons, nervous system, MSC

## Abstract

Cellular communication and the transfer of information from one cell to another is crucial for cell viability and homeostasis. During the last decade, tunneling nanotubes (TNTs) have attracted scientific attention, not only as a means of direct intercellular communication, but also as a possible system to transport biological cargo between distant cells. Peculiar TNT characteristics make them both able to increase cellular survival capacities, as well as a potential target of neurodegenerative disease progression. Despite TNT formation having been documented in a number of cell types, the exact mechanisms triggering their formation are still not completely known. In this review, we will summarize and highlight those studies focusing on TNT formation in the nervous system, as well as their role in neurodegenerative diseases. Moreover, we aim to stress some possible mechanisms and important proteins probably involved in TNT formation in the nervous system.

## 1. Introduction

### 1.1. TNTs as a Novel Means for Cell Interaction

Communication between cells is fundamental for maintaining homeostasis in multicellular organisms. It occurs through a variety of mechanisms: distant cells can deliver some secreted signaling molecules to each other; instead, adjacent cells use junctional complexes such as connexin-formed gap junctions (GJs) and synaptic junctions. In addition to these well-established pathways, tunneling nanotubes (TNTs) between cells have emerged as a means of long-distance communication, without the involvement of soluble factors [1]. TNTs are described as non-adherent actin-based cytoplasmic extensions that, as thin membranous bridges, connect cells over long distances and represent a novel direct method of communication. Rustom et al. (2004), who first described the yet unknown type of ultrafine intercellular connections, referred to as TNTs, which are formed a few hours after cell plating. They are very similar to another kind of membrane bridges, named cytonemes, described as filopodia-like structures connecting cellular membranes [2,3]. However, cytonemes have been reported to serve as tracks for membrane-associated molecules, which moved outside them, while TNTs are able to transfer inside them cytoplasmic materials [2]. In particular, Rustom and colleagues demonstrated that these connections contain F-actin and not microtubules, and are able to translocate endocytic vesicles through them. Moreover, these authors observed that the transfer had a unidirectional flow and it was found in different cell types, thus hypothesizing that TNTs represent a general cellular phenomenon important for long distance cell-to-cell communication [4].

The other authors then observed that their formation was not restricted to pairs of cells, but could lead to complex cellular networks. Subsequently, different publications described TNTs as important for intercellular exchanges of signals, molecules, and organelles, and are thus involved in different cellular functions. TNTs were extensively studied in epithelial cells [5], renal tubular cells [6], cardiomyocytes [7], and immune cells [8]. Lately, TNT communication has also been confirmed between neuronal cells [9,10,11]. On the other hand, it has been shown that TNTs could be involved also in pathological events, such as the propagation of pathogens and viruses. Different types of viruses have been reported to trigger the formation of TNTs and spread from infected cells to uninfected cells [12,13].

Until now, TNTs have been extensively described in simple monolayers of primary cells or cell lines. Although they have also been found in more complex systems, such as between cells within human mesenchymal stem cell (MSC) spheroids, consistent in size and geometry with those observed in the cellular monolayer culture, where they resulted in being involved in the exchange of the cytoplasm among connected cells [14]. Moreover, TNTs have been also found in tumor spheroids [15], thus hypothesizing that they may contribute to tumor survival and progression [16]. So far, there is limited evidence that these structures exist in vivo in complex tissues. This has raised the question of whether TNTs may in fact be purely an in vitro phenomenon.

Few recent studies have described TNTs in vivo, in more complex mammalian tissue. Membrane nanotube-like structures have been noted in bone marrow derived MHCII+ cells in the corneal stroma, forming distinct intercellular bridges between dendritic cells [17]. Nevertheless, several studies have suggested the presence of TNT-like bridges in vivo during certain developmental processes, such as closure in the midbrain during neurulation in mice [18] or Drosophila [19], as a mechanism for the accurate alignment of epithelial sheets.

### 1.2. TNTs Characteristics

So far, no TNT-specific markers have been identified, and for this reason, morphological features remain the main criteria for TNT identification. TNT length can be variable, but it is mostly reported to be about 100–200 μm or less [20,21]. Moreover, TNT lengths can vary due to changing cellular distances during migration, indicating that TNT length can be dynamically regulated. Because of this, the diameter of TNT is often an alternative characteristic for TNT identification. Based on diameter, TNTs can be divided into two groups: nanotubes less than 0.7 μm in diameter, mainly containing actin and carrying just some portions of plasma membrane between cells, or nanotubes larger than 0.7 μm, containing both microfilaments of actin and microtubules, able to also carry larger components of the cytoplasm between cells, such as vesicles, mitochondria, and other organelles [4].

There are two alternative hypotheses concerning TNT formation, with the first one suggesting that TNTs could grow and extend similar to filopodia. Usually, such protrusions appear in bunches of several tubes that dynamically seek connections between the neighboring cells. The second hypothesis proposes that two adherent cells progressively pull apart, but keeping some filamentous connections, that are TNTs [2,22,23]. As TNTs are formed from cytoskeletal filaments of F-actin, spanning uniformly along their full length, actin is an important labeling target in TNT imaging. Moreover, F-actin-associated myosin motors are considered to be involved in the transport of cellular components inside TNTs. Furthermore, in F-actin, microtubules have also been detected in TNTs in some cells, such as immune cells, between primary neurons and astrocytes, and in HUVEC cells during cancer-induced angiogenesis, although their role and relevance in TNTs need to be clearly established [24].

**Figure 1:**
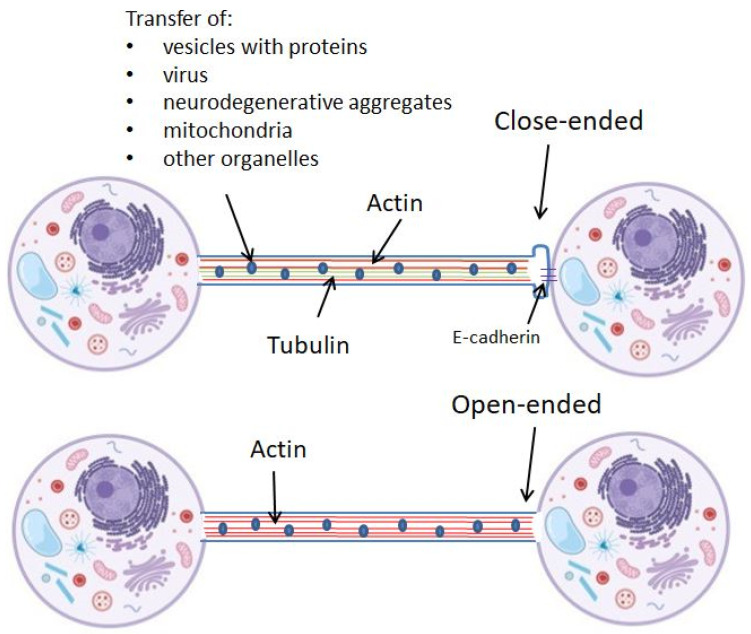
Schematic representation of two types: close-ended and open-ended TNTs. TNTs are built up from actin or both actin and tubulin filaments.

To act as a bridge between cells, the end of the TNT structure can form a junctional connection with the targeted cell, thus forming the close-ended TNTs, or it can connect the cell cytoplasms, by the so-called open-ended TNTs. Figure 1 schematically represents two types of TNTs.

The speed of TNT-dependent communication is mainly dependent on the types of transport. The transduction of electrical signals via TNTs is noted to be milliseconds, while the transport of organelles is dependent on motor proteins [25], it has been suggested to be associated with rapid rate TNT polymerization (0.2 μm/s) [26], and reported to be from 0.1 to 15 µm/s [27,28]. However, the spread of protein aggregates such as extracellular amyloid beta in TNTs is shown to be 2–8 times faster than ER, mitochondria, Golgi, and endosome transfer [28]. For example, the transfer of Tau aggregates in TNTs is reported to move with a speed of 2.83 ± 1.99 μm/min [29]. The transport of the virus by TNTs showed an average speed of 0.08 ± 0.03 μm/s, which is approximately 2–5 times faster than the movement of the murine leukemia virus along filopodial connections between cells [20]. This suggests that the transportation of biologic material by TNTs has different mechanisms, which can support the idea of different types of TNTs and their properties.

**Figure 2:**
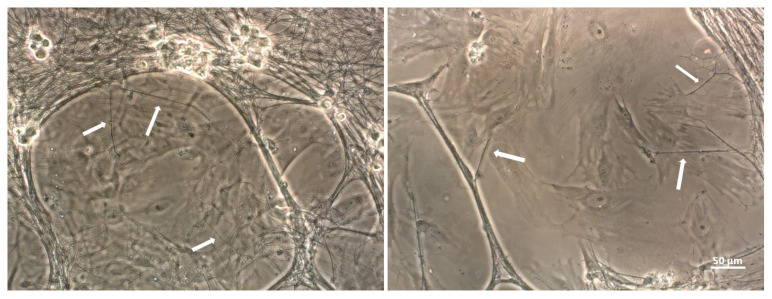
TNTs (white arrows) between MSCs and neurons observed in light microscopy.

While the majority of studies on TNTs have been observed between cells of the same population, recently, more attention has been given to the aspect of TNT formation between different cell types in co-culture [30], making them the important point of interest in both physiological and pathological conditions. Figure 2 represents TNTs formed in co-culture between neurons and MSCs (indicated with white arrow). In this review, we would like to highlight exclusively those articles that studied TNT formation between cells in the nervous system and their role in neurodegenerative diseases, in order to create a complete picture of all of the available information of TNTs in the nervous system. Neurons, different from other cells, have special abilities for long distance communication among them in networks, via electrical and chemical signals. The understanding of how TNTs are formed in the nervous system is of great importance, as they may have a direct role in neuronal interplay and cellular function.

## 2. Role of TNTs in Nervous System

### 2.1. Physiological Role: Neurogenesis

TNT formation seems to be important for neurogenesis. Cell-to-cell contact via long “thin filopodia” was observed between migrating neural crest cells (NCCs). It was found that NCCs maintained nearly constant contact with other migrating neural crest cells by short thin filopodia (about 1–2 µm) for local contacts or long-distance contacts (up to 100 µm). TNT length could be extended and retracted between two non-neighboring cells. Interestingly, the cell-to-cell contacts often stimulated a cell to change direction in favor of a neighboring cell’s migration, suggesting a possible role for these contacts in the coordination of NCCs directional guidance [31]. Similarly, the physiological role of TNT-dependent electrical coupling could be important for the synchronization and coordination of the migratory activity of NCCs within the expanded tissues during embryogenesis. It has been shown that TNTs are able to form bridges across the gap during neural tube closure, and they play a role in neural tube closure in the midbrain in the mammalian embryo [18].

On the other hand, it has been suggested that TNTs play a role in the electrical activity in early neuronal development. In this context, TNTs are hypothesized to transfer Ca^2+^, which is crucial for regulating the proliferation, migration, and differentiation of neurons [32]. Immature hippocampal neurons generated short protrusions towards astrocytes resulting in TNT formation. About 35% of immature neurons were electrically coupled with distant astrocytes via TNTs up to 5 h after co-culture, but not after 24 h. TNTs mediate the propagation of both depolarization and transient calcium signals from distant astrocytes to neurons. This suggests that, within a limited maturation period, developing neurons establish electrical coupling and exchange of calcium signals with astrocytes via TNTs at the first stage of maturation [33].

The hypothesis of electric signal transmission by TNTs in neurons during neurogenesis is also supported by Wang et al. (2010). Using optical membrane-potential measurements combined with mechanical stimulation and the whole cell patch-clamp technique, it has been shown that TNTs mediated the bidirectional spread of electrical signals between TNT-connected NCC cells, and this depends on the expression of connexin 43. PC12 cells that do not express gap junctions showed no electrical coupling by TNTs, which suggests that there are at least two different types of TNTs: those that retain connexins and participate in electrical coupling, and those that lack connexins and do not display electrical coupling. This finding suggests that different mechanisms of intercellular calcium signaling likely exist and may reflect the diversity of TNT structures and functions in specific cell types [34].

Examining the fundamental properties of TNTs, it can be concluded that, although in adult tissues, TNTs are stimulated by different diseases, stresses, and inflammatory signals, during neurogenesis, TNTs could play another role. Based on the similarity of the processes of synaptic spine development and TNT formation, it has been suggested that TNTs are involved in neuronal development and are involved in electrical signals transmission, as an important mechanism in immature neuronal circuits [35].

### 2.2. Pathological Role: Aggregates Propagation

Most neurodegenerative diseases are characterized by the accumulation of protein aggregates, some of which are toxic to cells. A large number of publications have described that TNTs could be involved in the propagation of aggregated proteins between neurons in neurodegenerative diseases. The ability of misfolded α-syn to aggregate and spread throughout the brain has important implications for Parkinson’s disease (PD) progression. It has been shown that human iPS cell-derived neuronal precursors (hNPCs) differentiated towards the dopaminergic lineage could have an active role in the internalization and propagation of α-syn fibrils, which can be transferred between hNPCs by TNT-like structures [36], thus suggesting that α-syn fibrils can be transferred through TNTs inside lysosomal vesicles. Donor cells, overloaded with α-syn damaged lysosomes, would transfer into healthy cells by hijacking the trafficking of TNT-mediated lysosomal vesicles [37]. The propagation of α-syn was also identified in microglial cells, which can form a functional network of F-actin-positive membrane connections of various lengths and diameters: shorter and thicker connections could be responsible for the transfer of large α-syn aggregates within 40–60 min, while longer and thinner connections transferred small α-syn aggregates between the microglia in about 3 min. It has been suggested that α-syn induced cytoskeletal changes and the formation of a TNT network resulted in an efficient reduction in the total α-syn [38]. In addition, the polyglutamine (polyQ)aggregates in Huntington’s disease are associated with TNT mediated transfer [39]. It has been shown that exogenously added Tau species triggered TNT formation between primary neurons, thereby facilitating the intercellular propagation of Tau fibrils, thus suggesting that Tau may promote the formation and function of highly dynamic TNTs [29]. However, whether TNTs contributes to aggregate propagation in vivo in the same way remains to be established.

Different hypotheses try to explain the role of TNTs in aggregate propagation. It has been suggested that the extracellular addition of protein aggregates could represent a source of stress and a pathological condition, and may likely act as a signal for TNT formation. TNTs may represent a common “rescue” mechanism adopted by the cell to get rid of toxic materials, such as protein aggregates or damaged organelles. An alternative hypothesis could be that stressed cells emit TNTs to be rescued, and that the aggregates = simply exploit these passages to transfer and spread between cells [40].

Remarkably, the inhibition of the formation of TNTs reduces intercellular transfer of aggregates [37,39,40], thus making TNTs a potential target for the treatment of neurodegenerative diseases. On the other hand, TNTs could also be exploited for the programmed transfer of drugs, as observed in microglial cells, which could mediate efficient drug transportation to glioma cells via TNTs and EVs [41], underlining the important therapeutic application of these structures.

### 2.3. Protective Role: Mitochondrial Transfer

Nowadays, one of the most widely studied aspects in the literature is the role of TNTs in the transfer of the mitochondria. Mitochondrial malfunction, accumulation of mtDNA mutations, and a reduction in respiratory chain activity, associated with an increase in downstream oxidative stress, can reduce cellular bioenergetics, and thus they are considered as important factors involved in neurodegeneration. The mitochondria not only supply the energy for cell function, but also take part in cell signaling. Moreover, because mitochondrial activity is required to maintain lysosomal structure, their dysfunction can influence lysosomal activity, leading to the accumulation of ubiquitinated protein aggregates [42,43] such as α-Syn in Parkinson’s disease [44] or amyloid-β (Aβ) proteins in Alzheimer’s disease [45].

Thus, drugs that improve mitochondria function could also regulate excessive ROS, thus having the potential to counteract neurodegenerative diseases. Because of the discovery of mitochondrial transport by TNTs, many studies have analyzed this aspect in different cellular models as a basis for cell therapy with the aim to replace malfunctioning organelles. The in vitro transfer of the mitochondria to different cell lines could recover mitochondrial functioning, rescue damaged cells, and support cellular survival. TNTs are among the mechanisms that allow MSCs to transfer mitochondria and prosurvival biomaterials, thus promoting tissue regeneration [46,47,48]. For example, the transfer of mitochondria from MSCs to damaged PC12 cells has been shown. PC12 has an embryonic origin from the neural crest and they are used as neuronal cell model due to their ability to acquire the sympathetic neuron features when treated with nerve growth factor (NGF). Co-culture with MSCs significantly reduced apoptosis and restored the membrane potential in injured cells. Mitochondrial transfer from MSCs to PC12 cells has been observed via formed TNTs. More importantly, the inhibition of TNTs partially abrogated the beneficial effects of MSCs on injured PC12 cells. MSCs reduced PC12 cell injury and these effects were in part due to efficacious mitochondrial transfer [49].

Moreover, it has been demonstrated that MSCs are able to transfer the mitochondria to primary neurons. In particular, it has been demonstrated that MSCs and neurons in co-culture form different intercommunication structures, able to transfer cytoplasmic materials and mitochondria. Mitochondria were identified along TNTs that were migrating from MSCs to neurons and not vice versa [30].

## 3. Mechanism of TNT Formation in Nervous System

Although the role of TNTs in the nervous system and their involvement in neurodegenerative diseases has been extensively studied, the exact mechanism triggering TNT formation in the nervous system is poorly described. As mentioned before, TNTs could form by a direct outgrowth of filopodia-like protrusions, or as bridges keeping in touch, separating cells. However, concerning the nervous system, Bukoreshtliev et al. supported the first hypothesis, suggesting that the first intercellular bridges are established by an outgrowth of a filopodia-like protrusion, rich in cytoskeletal filaments, toward a neighboring cell. These authors showed that the selective elimination of filopodia from PC12 cells by cytochalasin B (CytoB) blocked TNT formation, although with a low influence on the stability of existing TNTs. At these conditions, the intercellular organelle transfer was strongly reduced, while endocytosis and phagocytosis remain unaffected. These findings supported that in PC12 cells, filopodia-like protrusions could be the principal precursors of TNTs, and CytoB provided a method to selectively interfere with TNT-mediated cell-to-cell communication. It has been suggested that, although TNTs originate from filopodia, they developed into F-actin-based bridges with a unique morphology and distinct properties [50].

Other authors have suggested that stressful conditions can induce TNT formation. Stressed cells are strongly influenced by intercellular communication networks. The relation between stress conditions and TNT formation has been analyzed by Wang et al. (2015). The authors studied the role of TNTs in the interaction between apoptotic and healthy cells and showed that PC12 cells after being stressed with ultraviolet light (UV) were rescued by untreated PC12 cells in co-culture. UV-treated cells formed TNTs with untreated PC12 cells, which were characterized by the presence of microtubules inside the TNTs. The transport of mitochondria in the TNTs formed by stressed cells has been noticed. Moreover, the rescue effect was inhibited by incubating with an F-actin-depolymerizing drug, or when the mitochondria of rescuer cells were damaged, thus confirming that functional mitochondria delivery by TNTs is important for PC12 cell recovery in the early stages of apoptosis [51]. Similarly, Babenko et al. (2018) explored the transfer of mitochondria from MSC to neural cells and analyzed their efficacy under both normal conditions and after the induction of mitochondrial damage. It has been shown that mitochondria were transferred from MSCs to astrocytes and neuron-like PC12 in a more efficient manner when the cells were exposed to ischemic damage associated with elevated ROS levels. Such transport of mitochondria restored the bioenergetics of the recipient cells and stimulated their proliferation [52]. Transport of mitochondria was also noticed from astrocytes to neurons, probably supporting the neuronal viability and recovery after stroke [53].

### Molecular Pathways and Proteins Important for TNTs

Analyzing the literature, relevant attention needs to be given to the articles trying to identify those key proteins important for TNT formation. As TNTs could play an important role in cellular rescue as well as in neurodegeneration, these proteins may become important targets for therapy.

It has been reported that the Wnt/Ca^2+^ pathway, an intracellular cascade that is involved in actin cytoskeleton remodeling, has a role in TNT formation and TNT-mediated transfer of cargoes. As TNTs could be derived from the extension of filopodia-like protrusions toward neighboring cells, a process in which actin polymerization plays an important role. TNTs and filopodia use the same actin modulators and Rab proteins for their formation, so factors likely influencing filopodia formation could also be involved in TNT formation. Specifically, it has been found that Ca^2+^/calmodulin-dependent protein kinase II (CaMKII), a transducer of the Wnt/Ca^2+^ pathway, regulates TNTs in a neuronal cell line and in primary neurons. They identified the β isoform of CaMKII as a key molecule modulating TNT formation and transfer, showing that this depends on the actin-binding activity of the protein. Based on these findings, the following model has been proposed for the sequence of events underlying TNTs formation. Firstly, as result of stress stimuli, the appropriate Wnt ligand induces the phosphorylation of CaMKII. Phosphorylated βCaMKII detaches from actin. The loss of interaction between βCaMKII and F-actin could allow for the access of actin regulator proteins to the actin filaments. This would lead to an increase in actin polymerization events, with the consequent increment in the formation of TNT structures. After dephosphorylation, βCaMKII reattaches to F-actin, increasing the stability of the formed TNTs [9].

Furthermore, Tseng et al. (2021) also showed that Miro1 and TNFAIP2 are required for TNT formation and neuronal survival. Both Miro1 and TNFAIP2 expression were increased in the primary neurons after hydrogen peroxide exposure. The authors demonstrated the mitochondrial transfer from MSCs to neurons after oxidative stress within a relatively short (several hours) period. Moreover, they observed an improvement in survival, ATP production, and overall cellular metabolism that was dependent on cell-to-cell contact [54].

Rustom et al. (2004) analyzed endocytic vesicle translocation through TNTs in PC12 and reported that the vesicles moved in one direction only, with a speed in the range of actin-dependent transport. The authors showed that TNTs contained only F-actin and no microtubules, and that motor protein myosin Va was detected in these structures and has a role in facilitating organelle transport. Organelle exchange by TNTs was blocked after treatment with the F-actin depolymerizing substance latrunculin-B, thus strongly supporting actin and myosin as key elements for TNT formation [4]. Similarly, Gousset et al. (2013) observed that myosin 10 (Myo10), an unconventional motor protein that is usually associated with the filopodia, promotes TNT formation by interacting with several transmembrane proteins. Myo10 protein expression increases the number of TNTs and vesicles transferred among the co-cultured cells. It has been suggested that Myo10-dependent dorsal filopodia are the precursors of TNTs in neuronal cells [10]. Going deeper, Delage et al. (2016) analyzed the regulatory complexes of actin, which play a role in TNT structure formation. It has been demonstrated that the filopodia-promoting CDC42/IRSp53/VASP network negatively regulates TNT formation and decreases TNT-mediated intercellular vesicle transfer in neuronal CAD cells [55]. This finding differs from the fact that in non-neuronal cells, the CDC42 activity is required for contact-dependent intercellular transfer [56], thus suggesting a cell type-dependent mechanisms of TNT formation [55].

The transfer of the mitochondria by TNTs has been widely described in the literature, in particular, different studies have demonstrated that mitochondrial transfer is dependent on the levels of Miro1, a mitochondrial Rho-GTPase that regulates intercellular mitochondrial movement. Miro1 is the first protein shown to accelerate mitochondrial transfer. The discovery of Miro1 as a pivotal element in the intercellular transport of the mitochondria gave rise to speculations about the possibility of targeted modifications of stem cells in order to enhance their donation of mitochondria and thus to increase their therapeutic efficacy. Different studies have demonstrated that a higher efficiency of the mitochondria transfer after the overexpression of Miro1 was associated with enhanced positive effects of MSC [26]. In addition, in neuronal cells, Miro1 has been noted as an important player. The electrogenic activity of neurons requires cellular energy and high ATP consumption. The mitochondria provide over 90% of the total cellular ATP via oxidative phosphorylation. The localization of the mitochondria within a neuron is important for cellular ATP capacity and for the dynamics of intracellular Ca^2+^ signaling. Neurons are highly polarized and the length of their axons, mitochondrial transport, and signaling are crucial for cellular functionality [57]. An alteration of Miro1 function was found to play a role in the incidence of pathologies such as Parkinson’s disease [58], amyotrophic lateral sclerosis [59], and schizophrenia [60]. This hypothesis was also supported by Babenko et al. (2018), who showed that the use of MSCs overexpressing Miro1 in animals exposed to experimental stroke led to the significantly improved recovery of neurological functions. They hypothesized a key role of Miro1, which could promote the mitochondrial transfer from MSCs and suggested that the genetic modification of stem cells could improve therapies for the injured brain [52].

Extensive attention needs to be given to another publication, where p53 was analyzed as an important player for TNT formation. Wang et al. (2011) showed that TNT formation can be induced in rat hippocampal astrocytes and neurons in stress conditions, and the stressed cells are those that develop TNTs towards unstressed cells. It has been suggested that p53 is crucial for TNT development. p53 is suggested to regulate and interact directly with F-actin during TNT development. They proposed that cellular stress condition induces p53 activation as a mechanism, which then in turn upregulates the EGFR expression and activates further downstream pathway proteins Akt, phosphoinositide 3-kinase, and mTOR. The latter induces M-Sec overexpression, which, together with RalA and the exocyst complex, can trigger F-actin polymerization and induces TNT development. It has been shown that TNT development is inhibited when p53 is silent. Furthermore, it has been demonstrated that TNT uni-directional transport of cellular substances began from the damaged cell, hypothesizing that TNTs play a role in selective and targeted interaction between cells as a potential defense response to stress [28]. However, it is important to mention that the role of p53 in TNT formation has been argued by Andresen et al. (2013). In this publication, the authors investigated whether p53 is a key protein needed for TNT formation in PC12 cells. They demonstrated that TNT formation is cell-type dependent and p53 independent [61]. Considering the broad effect of p53 on numerous cellular functions, its role in TNT formation still needs to be confirmed.

Furthermore, Sun et al. (2012) identified S100A4 as an extracellular attractive molecule, which has a role in direction guidance during TNT formation. S100A4 belongs to a calcium-binding S100 protein family with at least 21 other members, localized in the cytoplasm, nucleus, and extracellular space. S100A4 has been extensively studied for its role in metastasis through its implication in angiogenesis, invasion, and cell motility. Although the majority of extracellular proteins are secreted by the endoplasmic reticulum (ER)/Golgi-dependent secretory pathway, increasing data shows that a group of nonclassical secreted proteins, such as S100A4, are exported through an ER/Golgi-independent pathway. S100A4 mediates TNT guidance through RAGE, a putative cell surface receptor for S100A4, in initiating cells. In particular, it has been proposed that the extracellular S100A4 attracts TNTs and thus has a role in TNT direction guidance. In the stressed initiating cells, p53 activation induces TNT development as well as caspase-3 activity. Caspase-3 cleaves intracellular S100A4, which therefore creates a chemical gradient around the target cells by inducing a relatively high concentration of S100A4. TNTs then follow this gradient to find the target cells [11].

In Table 1, we summarize some important publications on TNT formation in the nervous system.

## 4. Conclusions

Nowadays, extensive research is focused on cell-to-cell communications and their influence on cellular behavior and functions. TNTs represent a new emerging cell-to-cell communication tool, allowing for the transfer of the cellular components and molecules between cells over long distances. Different studies have suggested that TNT formation could be induced by stressful conditions. It has also been noticed that in the nervous system, TNTs have been shown to be involved in electric signal conduction, but only in the early stage of development. Most in vitro studies showed that TNTs seem to play a role in molecule and organelle transfer, but, at the same time, it is emphasized that it is still unclear whether the same mechanism works in vivo. The knowledge of the molecular mechanisms underlying TNT formation and the mechanisms of transfer of cellular components between cells in vitro and in vivo might provide further information regarding these physiological and pathological processes. The positive effect of MSCs on cell survival mediated by TNTs would be of particular interest in non-replaceable cells such as adult neurons. Moreover, understanding the role of TNTs in neurodegenerative diseases may provide new approaches to block misfolded protein aggregates and their spread. Despite awareness of the potential molecular mechanisms, the specific molecular markers and the signaling pathways involved in the initiation of TNT formation, destination, and stabilization are still not completely understood. In this review, we discussed publications that studied TNT formation in the nervous system and highlighted a few important molecular players; however, further studies are required to clarify the specific markers and the complex molecular network behind TNT formation.

## Figures and Tables

**Table 1:** Summary of main articles that analyzed TNT formation in the nervous system.

Cell Type	Described Mechanism	Observation	Conclusion	Important Molecule	Ref.
PC12	TNT formation in normal conditions	Block of TNTs formation caused the arrest of organelle transfer	Filopodia are the main precursor of TNTs that further develop into F-actin structures	F-actin	[50]
PC12	Rescue from UV stress by TNTs	Stressed cells formed TNTs formation and mitochondria transfer Stressed cells showed very early apoptotic signs, but without caspase-3 activation	TNTs are formed in the cells with damaged mitochondria at early apoptotic stages (before caspase-3 activation)	EB3	[51]
PC12, astrocytes	Mitochondria transfer to astrocytes or neuronal cells	Stressed cells formed TNTs formation and to transit mitochondria transfer. Overexpressed Miro1 in MSC improved neurological recovery after stroke	Miro1 is important for the transport of mitochondria in neural cells	Miro1	[52]
neurons, astrocytes	Transfer of mitochondria from astrocytes to neurons	Mitochondria transfer from astrocytes to neurons increased neuronal survival in transient focal cerebral ischaemia mice	Astrocytes release extracellular mitochondria via CD38-mediated mechanisms. Integrin-mediated Src/Syk signaling may be involved	CD38	[53]
CAD cells	TNT formation in normal conditions	CaMKII regulated TNTs formation. Wnt5a and Wnt7a significantly increased TNTs connections. Wnt7 affected TNTs formation, but not vesicle transfer	Wnt/Ca^2+^ pathway is involved in actin cytoskeleton remodeling, regulated TNT formation and the transfer of vesicles and α-synuclein aggregates	β isoform of CaMKII, Wnt5a, and Wnt7a	[9]
neurons	Transfer of mitochondria from MSC to neurons in stress conditions	Stress conditions increased Miro1 and TNFAIP2 expression Miro1 overexpression in MSCs increased mitochondrial transfer	Transfer of mitochondria to oxidant-damaged neurons may help improve neuronal survival and functional recovery after stroke	Miro1 and TNFAIP2	[54]
PC12	TNT formation in normal conditions	TNTs increased in the first 2 h of culture. Myosin Va was present inside TNTs and facilitated organelle transport	TNTs are formed as complex cellular networks. Actin is the major structural component of TNTs	actin and myosin	[4]
CAD cells	TNT formation in stress conditions	Myo10 overexpression results in the formation of functional TNTs and increased vesicle transfer between cells	TNTs can arise from a subset of Myo10-driven dorsal filopodia, independent of their binding to integrins and N-cadherins	Myo10	[10]
CAD cells	TNT formation in normal conditions	Activation of CDC42/IRSp53/VASP network negatively regulates TNTs formation and vesicle transfer. Eps8 increases TNT formation	Although TNTs and filopodia are completely different, the same actin regulators may be involved in their formation	CDC42, IRSp53, VASP, IRSp53, and Eps8	[55]
neurons, astrocytes	TNT formation in stress conditions	Stress induced p53 activation, which in turn upregulated EGFR expression and activated downstream pathways stimulating TNT formation	p53 regulates and directly interacts with F-actin during TNT development	p53, Akt, PI3K, and mTOR	[28]
neurons, astrocytes	TNT formation in both normal and stress conditions	p53 activation induced TNT formation through the activation of caspase-3. Activated caspase-3, by acting on S100A4, created a chemical gradient to direct TNT formation	Extracellular S100A4 directs TNT direction formation and, thus, has a role in TNT guidance	S100A4	[11]

## Data Availability

Not applicable.
